# Public Pharmacists' Association

**Published:** 1918-02-23

**Authors:** 


### Public Pharmacists' Association.

The annual general meeting of this Association was held at St. Bride Institute, E.C., on Wednesday, February 6, 1917, Mr. R. Welford (Chairman of the Association) presiding.

The annual report of the Council explained that this is the only Association which represents pharmacists engaged in instititutional work solely, and that, in view of the many proposed changes in the treatment of the sick, it was important that united action should be taken to secure for public pharmacists a place in the medical treatment of the sick poor.

New officers were elected as follows: President, T. H. W. Idris, Esq., J.P.,; Chairman, A. H. Jenkin; Vice-Chairman, A. Howell; Hon. Sec. and Treasurer, Geo. W. Gibson; Hon. Sec. "Poor-Law Section," R. Lindsey. Council: Miss Andrews (Paddington), Messrs. Gibbons (St. Thomas's), Welford (L.C.C.), Windmill (H.M. Prisons), Skinner (G.N.C.), France (Westminster), Moore (St. Pancras), H. C. Thomas (Wandsworth).

It was agreed to ask the M.A.B. (pharmacists) to nominate a representative—thus making an executive to represent practically all branches of institutional dispensing.

Mr. R. Welford, in retiring from the chair, referred to the past work of the Association, and the advantages, social, educational, and material, obtained by combination of the various branches of pharmaceutical workers engaged at institutions, and pleaded for a more generous support from the general hospitals. Mr. A. H. Jenkin, the new Chairman, referred to the problem of dealing with partially trained dispensers who had not taken the qualification of "pharmacist."

Mr. S. J. Elliott (Southport Infirmary) contributed a paper on "Eusol," and outlined a method using a concentrated chlorine solution "Chloros," which rendered very satisfactory results. Mr. Elliott also gave a formula for a "hand" solution, to remove the objectionable smell left after using "Eusol," this being a solution of sodium hyposulphite in water. The paper gave rise to a good debate on this and kindred antiseptic solutions.

